# Scottish Vision Group, Carnoustie, Scotland, UK, 20–22 March 2015

**DOI:** 10.1177/2041669515593223

**Published:** 2016-02-29

## Abstracts

**15th Annual Meeting**

Ken Scott-Brown

Alasdair Clarke

George Lovell, Nick Wade, Anita Simmers

Previous meetings have been held at:

2001 The Burn, Glenesk, Brechin, Angus

2002 The Burn, Glenesk, Brechin, Angus

2003 The Burn, Glenesk, Brechin, Angus

2004 The Burn, Glenesk, Brechin, Angus

2005 The Log Cabin Hotel, Kirkmichael, Perthshire

2006 The University of Aberdeen, Aberdeen

2007 Kinloch Hotel, Blackwaterfoot, Isle of Arran

2008 Dundarach Hotel, Pilochry, Perthshire

2009 Western Isles Hotel, Tobermory, Isle of Mull

2010 The Royal Dunkeld Hotel, Dunkeld, Perthshire

2011 Sabhal Mor Ostaig, Sleat, Isle of Skye

2012 Douneside House, Tarland, Royal Teeside

2013 Ballachulish Hotel, Ballachulish, Glencoe

2014 The Marine Hotel, Troon, South Ayrshire

2015 The Carnoustie Golf Hotel, Carnoustie, Angus

## Solving the Aperture Problem of Binocular 3D Motion Perception

**M Lages and S Heron**

University of Glasgow, UK

**Abstract**

Motion transparency, the perception of distinct, overlapping patterns of motion, may be observed when independent random-dot patterns move over one another in opposing directions. Observers perceive such stimuli as separately moving surfaces, with each surface ordered in depth. Several factors affect this perception of depth-order, including dot size, speed and density (Schutz, 2012), with individualised directional biases in depth-ordering also noted (Mamassian in anticorrelated adaptation, each eye received the photographic negative of the other eye's image, which selectively adapted the differencing channel. These adaptation stimuli had equal energy at all orientations. Despite being isotropic, the adaptors influenced perceived tilt: The test stimulus usually appeared tilted in the difference direction after correlated adaptation, and usually appeared tilted in the summation direction after anticorrelated adaptation. This counterintuitive finding of a tilt aftereffect from isotropic adaptors is exactly analogous to May, Zhaoping and Hibbard's (2012) finding of a motion aftereffect from static adaptors. These two results strongly support Li and Atick's theory.

## Perception of Depth-Order in Motion Transparency

**R Goutcher**

Division of Psychology, School of Natural Sciences, Univ. of Stirling, UK

ross.goutcher@stir.ac.uk

**Abstract**

Motion transparency, the perception of distinct, overlapping patterns of motion, may be observed when independent random-dot patterns move over one another in opposing directions. Observers perceive such stimuli as separately moving surfaces, with each surface ordered in depth. Several factors affect this perception of depth-order, including dot size, speed and density (Schutz, 2012), with individualised directional biases in depth-ordering also noted (Mamassian in anticorrelated adaptation, each eye received the photographic negative of the other eye's image, which selectively adapted the differencing channel. These adaptation stimuli had equal energy at all orientations. Despite being isotropic, the adaptors influenced perceived tilt: The test stimulus usually appeared tilted in the difference direction after correlated adaptation, and usually appeared tilted in the summation direction after anticorrelated adaptation. This counterintuitive finding of a tilt aftereffect from isotropic adaptors is exactly analogous to May, Zhaoping and Hibbard's (2012) finding of a motion aftereffect from static adaptors. These two results strongly support Li and Atick's theory.

## When does an encoding become a percept?

**D W Hunter and P B Hibbard**

School of Psychology and Neuroscience, University of St Andrews

Department of Psychology, University of Essex

**Abstract**

How the visual signal is processed by early visual systems, from the retina to V1 has been extensively studied. Clear patterns have been observed that allows us to categorise different levels of processing. A substantial proportion of the functionality of retinal ganglion cells can be described as performing signal deconvolution and whitening using a centre-surround pattern (Graham, Chandler, Field 2006). V1 simplecells have been found that can be characterised as performing a linear convolution using a Gabor-like pattern (Olshausen, Field 2005). This has been shown to be consistent with the sparse energy efficient coding theory (Barlow 61) using a variety of metrics, e.g. Olshausen darker chromatic components matched with darker edges and vice versa. The smallest gradient width had the lowest perturbation thresholds. This supports the idea that chromatic edges and luminance lines have a specific relationship.

## The role of feedback in motor movements: Assessing the allocation of attention in goaldirected pointing and saccades

**A Mahon, C Hesse and A R Hunt**

University of Aberdeen, UK

r05am14@abdn.ac.uk

**Abstract**

Prior to executing a movement, attention is allocated to the movement target. However, when executing skilled actions, such as driving, performance is often improved when attention is directed to the external locations of feedback. In light of these findings we examined the role of feedback and attentional allocation in the planning and execution of both pointing movements and saccades. Participants were presented with a circular array of eight digital 8 s. They were asked to point or saccade towards a movement target, as indicated by a central arrow, while simultaneously identifying a briefly-presented discrimination target. Visual feedback on the accuracy of movement was provided, in the form of a brief color change in one of the 8 s immediately following the end of the movement. The results show elevated discrimination accuracy at both movement targets and feedback locations for pointing, but only at the movement target for eye movements. We will discuss these findings in light of how attention could compensate for the effect of eye movements on the retinotopic locations of behaviorally relevant information.

## Search strategies in simulated hemianopia

**A Nowakowska, A D F Clarke, A Sahraie and A R Hunt**

University of Aberdeen, UK

**Abstract**

During search, patients with hemianopia tend to direct eye movements toward the intact visual field, causing a larger proportion of the search array to fall within the damaged field. Rehabilitation programs often train patients to make large eye movements into their damaged field. We show that the effectiveness of this strategy depends on where and what kind of information is present in both the damaged and intact field. Healthy participants searched for an emotional face with half of their visual field degraded or removed. Participants were biased towards the sighted field over the blind, and the proportion of saccades directed towards the blind field increased with the amount of information available. However, searching the initially sighted field first was not associated with slower search. Shifting the location of the initial fixation point into the blind side, making the search array fully visible at the start of the trial, also did not improve search overall: The target was found faster when it was on the damaged side, but slower when it was on the sighted side, negating the benefit of starting search on the blind side.

## Does countershading impede efficient visual search?

**O Penacchio, P G Lovell, S Sanghera, I C Cuthill, G D Ruxton and J M Harris**

School of Psychology and Neuroscience, University of St Andrews, UK

School of Social and Health Sciences, Abertay University, UK

School of Biological Sciences, University of Bristol, UK

Centre for Biological Diversity, University of St Andrews, UK

**Abstract**

Shading is a central cue to shape perception in humans. There is evidence that other animals use shading to apprehend shape. If some predators are able to use shading to detect/evaluate preys, it makes sense to evolve strategies to conceal these cues. In nature more light comes from above. Therefore, to conceal shape-from shading a body should counterbalance the shadowing created by directional light. Such a pattern, called countershading, is darker where the body is usually exposed to a greater light intensity. Countershading colouration is widespread throughout the animal kingdom. We used a computational model that incorporates realistic lighting environments to determine what pattern of countershading yields optimal camouflage of 3D shape for different lighting conditions. We tested countershading experimentally in a visual search task with human observers. Target 3D objects with different levels of camouflage were displayed on a screen amongst flat objects. Participants had to detect the target object as quickly as possible. The experiment showed that countershading camouflage has a very significant effect on both detection speed and accuracy. A predation experiment with birds based on the same modelling gave similar results. Taken together, these experiments suggest that countershading obstructs efficient visual search and reduces visibility.

## Following the line: Non-social dynamic cues automatically guide attention

**S E A Gregory and M C Jackson**

School of Psychology, University of Aberdeen

**Abstract**

Non-predictive shifts in eye gaze can orient attention rapidly and automatically. Arrows are traditionally used as a non-social, non-verbal cue comparison. However, arrow cues are problematic in that not only are they over-learned and frequently used, they are a static directional cue while eye gaze shifts involve motion. Arrows may therefore not be the best non-social control. To address this, we created a novel dynamic attention cue which is not social, not over-familiar, nor easily verbalised. The cue consists of a horizontal line centrally bisected by a shorter vertical line. This vertical line shifts either left or right thus reflecting the dynamic shift of a pupil and iris. Participants were required to locate an asterisk that appeared on the left or right of the horizontal line at a stimulus onset asynchrony (SOA) of 150/300/500/1000/2000 ms after the motion cue. At early SOAs the cue significantly speeded target detection in the cued versus uncued location, comparable to attention shifts reported in gaze cuing studies. This dynamic cue is thus a valuable tool which will allow researchers to better tease apart mechanisms of attention shifting that are related to the social nature of a cue versus low-level motion information.

## Attentional modulation of responses in human face- and voice-sensitive cortex

**Q C Vuong, Y Kikuchi, J Ip, J C Mossom, N Barraclough and C I Petkov**

Institute of Neuroscience, Newcastle University Medical School, UK Department of Psychology, University of York, UK

**Abstract**

Attention plays an important role in helping observers select relevant information for the task at hand. There is considerable interest in understanding how attention modulates neural changes in different brain regions. For example, attention can increase neural response magnitude or neural selectivity in regions that respond to the relevant stimuli. However, whether attention modulates neural changes similarly across different sensory modalities remains unclear. We addressed this question using fMRI repetition suppression in which the repetition of the same or similar stimuli lead to a reduction in the fMRI response. Twelve volunteers participated in comparable auditory and visual fMRI experiments in which they directed their attention to voice or face identity changes, or to spatial changes of the respective stimuli. Importantly, we equated performance on the identity and spatial tasks across both modalities. For functionally-localised face and voice regions of interest (ROIs), there was a significantly larger repetition effect when volunteers attended to identity differences relative to when they attended to spatial differences. Moreover, attentional modulation was specific to face/voice-sensitive cortex because it was not evident in regions outside of these ROIs. Overall, the results suggest that attention to stimulus features (e.g., identity) increases neural responses comparably across different sensory modalities. Supported by a Biotechnology and Biological Sciences Research Council grant to CIP and QCV

## Pupillometric Indices of Target Detection in a Signal Detection Task

**J T Martin and S Johnston**

School of Psychology, Swansea University Pupillometry is used widely in psychological research to infer mental processing. Specifically, pupil dilation has been linked to increased effort in extended wide-field visual search (Porter, Troscianko Computer Science Department, Universitat AutOEnoma de Barcelona

School of Psychology and Neuroscience, University of St Andrews

**Abstract**

The perceived intensity of an area is modified by the luminance of surrounding areas, a phenomenon known as brightness induction. Several phenomenological models have been proposed to account for the phenomenology of brightness induction effects. These models follow a multi-scale approach and sometimes integrate a contrast sensitivity function. In this study, we adopted a computational approach to examine potential neural mechanisms underlying brightness induction. Recent neurophysiological converging evidence suggests that brightness might be represented in the visual pathway as early as V1. We built up on a previous neurodynamical model of contextual modulation in V1 explaining contour integration and visual saliency (Li 1999), and added units tuned to different spatial frequencies. Both static and dynamic classical brightness induction effects emerged naturally from this architecture. We further tested the model for colour induction, a process whereby a colour is perceived differently depending on the proximity of other colours. This study suggests that contextual modulation in V1 could be, at least to a certain extent, responsible for brightness and colour induction effects. It also shows how a common general mechanism may account for several fundamental phenomena such as visual saliency, pre-attentive segmentation and brightness and colour induction effects.

## Utilising gaze training paradigms and cognitive tasks to train efficient visual behaviour when driving

**A K Mackenzie and J M Harris**

School of Psychology and Neuroscience, University of St Andrews

**Abstract**

We present two experiments that investigate potential training methods for drivers to learn more efficient visual behaviour when driving. The first experiment is a follow-up to Mackenzie and Harris (2014) where we looked at the effectiveness of an implicit gaze following technique by showing novice drivers the eye movement scan patterns of experienced drivers. Previously we found that after watching videos of expert drivers’ eye movements, novice drivers exhibited increased horizontal visual scanning of the road, larger saccade sizes and showed an increased use of their mirrors, during a simulated drive. Here we report the follow up results, where we find this visual behaviour is retained after a six month period. The second experiment aimed to identify potential attentional relationships between individuals’ cognitive ability and their visual performance when driving. We found that those who performed better on a battery of active cognitive tasks also exhibited more efficient visual behaviour when driving; as measured by increased horizontal scanning of the road, larger and faster saccades and more efficient use of interior and driverside mirrors. We suggest that training in active cognitive tasks may aid drivers with the cognitive demands of driving, which could allow more efficient visual behaviour when driving.

## Category effect in visual crowding = feature differences + overlap differences

**J Reuther and R Chakravarthi**

University of Aberdeen, UK

j.reuther@abdn.ac.uk

**Abstract**

Recognition of an object in the periphery is negatively influenced by other objects in close proximity. This crowding effect is modulated by the similarity between such adjacent objects. The more similar the objects features are (e.g., shape or spatial frequency), the stronger the crowding between the objects is. Recent evidence seems to suggest that this similarity effect extends to higher level properties of objects, such as their category membership: objects belonging to the same category crowd more than those belonging to different categories. In three experiments, we take a closer look at this alleged higher-level interaction in crowding while controlling for various low-level attributes. We find that the category effect is observed when there are featural differences between target and flankers or when there is substantial overlap between the two (target and flanker) stimulus sets; whereas the effect completely disappears when both are controlled for. We conclude that the effect cannot be attributed to object category, but can be reduced to low-level differences such as featural differences and differences in target- and distracter-set overlap. Based on thesefindings, we recommend caution when claiming the existence of higher-level interactions in crowding.

## Failure of intuition when presented with a choice between investing in a single goal or splitting resources between two goals

**A D F Clarke and A R Hunt**

University of Aberdeen, UK

a.clarke@abdn.ac.uk

**Abstract**

We face many competing demands on our attention in our daily life and regularly have to divide our time and attention between different tasks. Morvan darker chromatic components matched with darker edges and vice versa. The moderate gradient width had the highest perturbation thresholds. This may suggest that while lines and close to full field luminance edges facilitate perturbation detection, moderate width edges are not processed in the same manner.

